# Boosting Pharmacy Foundational Science Education Through Game-Based Learning and Active Engagement Strategies

**DOI:** 10.3390/pharmacy14040104

**Published:** 2026-07-09

**Authors:** Maria Victoria Tejada-Simón

**Affiliations:** Department of Pharmacological and Pharmaceutical Sciences, College of Pharmacy, University of Houston, 4349 Martin Luther King Blvd, Room 3044, Houston, TX 77204, USA; mvtejada@central.uh.edu

**Keywords:** game-based learning, pharmacy education, biochemistry, active learning, student engagement, health professions education, foundational science

## Abstract

**Background:** Game-based learning (GBL) and active engagement strategies have shown promise in health professions education; however, their application in foundational basic science courses, which serve as critical academic gatekeepers in professional pharmacy programs, remains underexplored. **Objective:** This study evaluated the effect of a multimodal suite of faculty-developed, web-based GBL activities and active engagement strategies on academic performance, learning management system (LMS) engagement, and student perceptions in a required first-year (P1) pharmacy biochemistry course. **Methods:** A cross-sectional, descriptive study was conducted with 117 P1 pharmacy students. Game-based activities (including Jeopardy, Rapid Fire, and Crossword Puzzle games) were developed using WiscOnline and deployed via Blackboard alongside pre-class activities, a Workbook, and a Padlet discussion board. Engagement was measured via LMS access metrics, academic performance via examination scores, and perceptions via an end-of-semester anonymous Poll Everywhere survey. **Results:** The cohort achieved a mean examination score of 81.59%, with 83.8% earning a passing grade. A positive association was observed between game access frequency and grade group performance (R^2^ = 0.8241), though individual-level correlations did not reach statistical significance. Student perceptions were overwhelmingly positive, with over 90% of respondents agreeing that GBL activities were enjoyable, facilitated learning, and provided valuable practice opportunities. **Conclusions:** Low-cost, faculty-developed GBL tools can be successfully integrated into foundational pharmacy science courses, yielding high engagement and positive student perceptions. These findings underscore the importance of extending active learning research to foundational basic science courses and offer a replicable model for health professions programs seeking to enhance student engagement at this critical curricular stage.

## 1. Introduction

Game-Based Learning (GBL) refers to the integration of game elements and principles into educational contexts to engage learners and enhance learning outcomes. It is grounded in constructivist educational theory, which views learning as an active process through which learners construct new knowledge based on experience and reflection [1,2], Historically, games have served as tools for developing strategic thinking long before the digital era. Chess, for example, was employed in military training as early as the seventh century [3]. The modern concept of GBL evolved substantially with the advent of digital technologies. Beginning in the 1980s, educational games gained widespread attention, with early titles such as *The Oregon Trail* introducing the concept of learning through simulation [3,4]. In the 21st century, increasingly sophisticated digital platforms and the integration of artificial intelligence have further transformed GBL, supporting personalized and adaptive learning experiences tailored to individual learner needs [2,5].

At its core, GBL environments facilitate learning by creating immersive, iterative experiences that encourage exploration, learning from failure, and progressive mastery. Its interactive architecture, incorporating elements such as challenge, immediate feedback, adaptive difficulty, and goal-directed progression, maps closely onto Bloom’s Taxonomy of educational objectives, scaffolding learners from foundational knowledge recall through higher-order cognitive processes, including application, analysis, and evaluation [6]. By leveraging the inherently engaging and interactive nature of games, GBL creates learning environments that foster critical thinking, problem-solving, and collaboration, competencies that are increasingly valued across academic disciplines and professional fields, including health care professions [7,8].

Traditional instructional methods in higher education, often centered on passive, lecture-based delivery, have been widely criticized for their limited capacity to sustain student engagement or promote deep conceptual understanding, particularly in cognitively demanding disciplines [7,9]. GBL addresses these limitations by providing interactive, immersive learning environments that encourage active participation, immediate feedback, and iterative problem-solving. Research has consistently demonstrated that GBL can lead to improved academic performance, higher levels of student satisfaction, and enhanced intrinsic motivation to learn [10,11]. Educational research has found significant positive effects of GBL on academic achievement across multiple subject areas [8], with consistent improvements in student engagement, motivation, and performance across STEM, social sciences, and language disciplines [9]. In language education specifically, researchers found that students learning through game-based platforms demonstrated superior vocabulary retention and higher engagement compared to those using conventional methods [12], a finding particularly relevant to content-heavy disciplines requiring extensive terminology acquisition, such as pharmacy.

In the context of pharmacy education, a field that demands mastery of a vast and complex body of knowledge alongside the development of sophisticated clinical reasoning skills, GBL offers particular advantages. Through simulations, role-playing scenarios, and problem-based game formats, GBL enables students to practice and refine skills in controlled, low-stakes environments that closely approximate the challenges of professional practice [1,6]. Notable examples include the *MyDispense* platform, a virtual pharmacy simulation developed by Monash University, which allows students to practice medication dispensing, prescription verification, and patient counseling in a realistic simulated workflow, with studies reporting improvements in practical exam performance and internship preparedness among users [1]. Similarly, the *Pharmacy Simulator* platform offers comprehensive environments encompassing patient counseling, therapeutic decision-making, and prescription management [6]. General-purpose platforms such as Kahoot and Quizlet have also been adapted for pharmacy education, offering flexible, interactive formats for reinforcing pharmacology content [11]. Emerging technologies, including virtual reality and augmented reality, are beginning to enter the pharmacy education landscape, with early evidence suggesting promise for enhancing learning outcomes. However, their adoption remains constrained by high development costs and specialized hardware requirements [2,8].

Despite these advances, the application of GBL in pharmacy education is not without limitations in both practice and the literature. High development costs, the need for faculty training in game design, platform sustainability concerns, and varying levels of student acceptance have been identified as barriers to widespread adoption [8,10]. Critically, the existing body of pharmacy GBL research has been concentrated primarily in advanced clinical and therapeutics courses, contexts where the relevance of game-based scenarios to professional practice is immediately apparent to students [6,11]. Far less attention has been directed toward the application of GBL in foundational courses and basic science courses, where the pedagogical challenge is distinctly different and, perhaps, more acute.

The first year of the PharmD program represents a critical inflection point in professional training. P1 students must rapidly assimilate a dense body of foundational science knowledge (including biochemistry, physiology, and related disciplines) that forms the conceptual scaffolding for all subsequent clinical coursework. In the health professions, courses at this foundational level frequently function as academic triage points, meaning that students who fail to achieve mastery of core principles face significantly elevated risk for academic difficulty in clinical transfer in advanced pharmacotherapy, medicinal chemistry, and clinical practice courses [13]. Unlike advanced clinical courses, where the connection between content and professional practice is explicit and immediately motivating, foundational biochemistry and related sciences require students to invest substantial cognitive effort in mastering abstract concepts whose clinical relevance may not become apparent until considerably later in the program. This delayed relevance, combined with high content density and the academic adjustment demands of the first professional year, creates conditions in which student disengagement and academic difficulty are particularly likely to emerge [14]. Intervening at this foundational stage with evidence-based active learning strategies is therefore not merely a pedagogical preference but a curricular imperative with downstream consequences for student success across the entire PharmD program.

While the existing literature provides robust evidence for GBL’s effectiveness in higher education broadly and in pharmacy clinical education specifically, several important gaps remain. First, the majority of pharmacy GBL research has focused on high-fidelity simulation platforms requiring substantial institutional investment, leaving the effectiveness of low-cost, faculty-developed game-based tools relatively understudied. Second, few studies have examined GBL implementation in foundational science courses within the PharmD curriculum, where abstract content and high cognitive load present distinct pedagogical challenges. Third, the relationship between voluntary, asynchronous GBL engagement (as measured by learning management system (LMS) access metrics) and summative academic performance has received limited empirical attention. Together, these gaps represent a missed opportunity, as the motivational and cognitive benefits of GBL may be particularly valuable precisely where student engagement is most difficult to sustain.

This study explored the relationship between a collection of faculty-developed, web-based GBL activities and academic performance in a required P1 pharmacy biochemistry course. Using freely available platforms, such as WiscOnline and Padlet, integrated directly into the institutional LMS, this intervention was designed to be both pedagogically effective and practically replicable by faculty at institutions without specialized instructional design support. Rather than seeking to establish a direct causal link, the study aimed to characterize how engagement with these tools serves as an indicator of the broader self-regulatory learning behaviors that underpin success in this required course.

Specifically, the study aims to: (1) describe student engagement patterns with game-based and supplementary learning resources as measured by LMS access metrics; (2) examine the association between resource engagement and examination performance; and (3) characterize student perceptions of GBL activities with respect to learning utility, enjoyment, confidence, and preference. By generating both empirical findings and a practical implementation model grounded in freely available tools, this study contributes to developing replicable, actionable insights to the growing literature on active learning and GBL in pharmacy education, with particular relevance to the foundational courses that serve as gatekeepers to professional competency in the PharmD curriculum.

## 2. Materials and Methods

### 2.1. Study Design and Setting

This study employed a prospective, descriptive, cross-sectional design to evaluate the effect of game-based learning (GBL) and other active engagement strategies on academic performance and student perceptions in a required first-year (P1) pharmacy biochemistry course. The study was conducted during the academic year 2022–2023 at the University of Houston College of Pharmacy (UH-COP). Because this was a single-course, cross-sectional, descriptive educational study, no a priori sample size calculation was performed. The study used a convenience sample consisting of the full available cohort of 117 P1 students enrolled in the required Biochemistry course during the Fall 2022 semester. Institutional Review Board approval was obtained (Study00006105), and all student data were de-identified before analysis to protect participant confidentiality.

### 2.2. Participants 

Participants included first-year pharmacy students enrolled in the Doctor of Pharmacy (PharmD) program at UH-COP. A total of 117 students participated in the study (Table 1), with a nearly equal distribution of male (48%) and female (52%) students. Participants ranged in age from 21 to 29 years, with a mean age of 24 years. All students were enrolled in Biochemistry as a mandatory component of the PharmD curriculum. This course was selected for the integration of GBL due to its conceptual complexity and the need for students to apply theoretical biochemical knowledge in clinically relevant scenarios. Participation was voluntary, and no incentives or rewards were offered to students beyond the educational value and enjoyment associated with engaging in the activities.

### 2.3. Instructional Interventions

A multimodal suite of active learning and game-based interventions was integrated into the Biochemistry–Metabolism module to supplement traditional didactic instruction. All resources were hosted and tracked through the learning management system (LMS) Blackboard (Anthology Inc., Boca Raton, FL, USA).

A. Game-Based Learning Tools (WiscOnline [currently called WisTechOpen])

Three game-based learning activities (Jeopardy [GI Vitamins and Minerals], Rapid Fire [Glucose Homeostasis], and Crossword Puzzle [Amino Acids]) were created using WiscOnline (Wisconsin Online Resource Center, Wisconsin Technical College System), a free, educator-developed platform designed specifically for the creation of interactive learning objects and educational games. WiscOnline offers a broad library of customizable game templates adaptable to virtually any academic discipline, making it a versatile and accessible tool for faculty across health professions education. A key feature of the platform is its ability to export newly created games as shareable content object reference model (SCORM) packages, which can be seamlessly embedded into any institutional LMS. This functionality allowed students to access and interact with the games directly within Blackboard without the need to create external accounts or navigate away from the course environment, thereby reducing access barriers and supporting integration into students’ existing study routines.

- Jeopardy—GI Vitamins and Minerals: A competitive, team-based Jeopardy-style game designed to reinforce content related to the gastrointestinal system, vitamins and minerals. The game was deployed both as an in-class activity and as an asynchronous self-study resource.
- Rapid Fire—Glucose Homeostasis: A rapid-response quiz game focused on glucose metabolism and its hormonal regulation, designed to reinforce recall and clinical application through high-frequency, time-pressured questioning.
- Crossword Puzzle—Amino Acids: An online crossword puzzle targeting amino acid nomenclature, classification, and biochemical properties, available as an independent asynchronous study tool.

It should be noted that after the completion of this study, WiscOnline transitioned to a restructured platform under the name WisTechOpen. During this migration, user-created content, including the game-based learning objects developed for this study, was subject to evaluation and removal. As a result, the specific games described herein are no longer available in their original form, and direct replication using the same resources is not possible. This transition underscores the importance of exporting and archiving SCORM packages locally at the time of creation, as institutional or platform-level changes may render cloud-hosted educational content inaccessible without prior backup.

B. Instructor-Developed Active Learning Activities

Two additional activities were developed directly by the course instructor to provide structured, low-stakes practice opportunities concurrent with course content delivery:

- Pre-Class Activities (GI Diagram): Fill-in-the-blank preparatory exercises designed to prime students for in-class instruction by reviewing gastrointestinal biochemical pathways. These activities were aligned with lecture content and available to students at any time for self-directed practice.
- Workbook: A structured digital Workbook containing fill-in-the-blank and practice problems aligned with module learning objectives. The Workbook was designed to run concurrently with lectures, offering students additional opportunities for content reinforcement and self-paced review at their discretion.

C. Discussion Board (Padlet)

An asynchronous peer-to-peer and faculty-facilitated discussion board was created using Padlet (Padlet Inc., San Francisco, CA, USA), a collaborative digital platform that allows users to post, respond to, and organize content in a visually accessible format. The discussion board served as a space for students to post questions, engage with peers, and interact with course content outside of scheduled class time.

D. Supplementary Resources

Study Help Folder: A curated collection of supplementary study materials, including summary sheets, annotated slides, and reference guides, accessible asynchronously through Blackboard.

### 2.4. Game Development Process

The game-based learning activities used in this study were developed by course faculty through a structured, iterative process grounded in alignment with course learning objectives. Rather than relying on external developers, all game content was created directly by the instructors using different platforms, ensuring fidelity between the game content/active learning activities and the specific learning outcomes of the Biochemistry curriculum. The development process followed these stages:

1. Conceptualization: Faculty identified the key learning objectives for each module and determined which content areas would benefit most from game-based reinforcement, prioritizing topics with high cognitive load or historically lower student performance.
2. Design: Faculty selected appropriate game formats from the template library (Jeopardy, Rapid Fire, Crossword Puzzle) and mapped course-specific content onto the game mechanics.
3. Development: Game content was authored within the WiscOnline platform, incorporating course-specific questions, answer sets, and feedback elements. Upon completion, each game was exported as a SCORM package.
4. Testing: Although the games were not formally validated in a separate pilot study, they were informally reviewed and tested by a small group of faculty before implementation to confirm functionality, clarity of instructions, alignment with course learning objectives, and usability within the LMS environment.
5. Implementation: Finalized SCORM packages were uploaded to Blackboard and embedded within the relevant course modules, making them immediately accessible to students within the existing LMS environment.

### 2.5. Game Integration into the Curriculum

The integration of game-based learning activities into the biochemistry curriculum was strategically planned to align with key content modules and reinforce specific learning objectives at appropriate points in the instructional sequence. Most game-based and active learning activities were made available asynchronously as supplementary learning tools through the LMS’s built-in access monitoring system, allowing students to access and complete them at their own pace. When used during class sessions, approximately 20 min of class time were typically devoted to game-based activities. Engagement analyses in this study were based on LMS access frequency rather than duration of use or time-on-task.

The games were not assigned a fixed number of times per week. Rather, they were continuously available through the LMS for voluntary, asynchronous use, and students accessed them according to their individual learning needs. Frequency of use was captured through LMS access metrics.

Detailed specifications for the design, implementation, and content of the Jeopardy, Rapid Fire, and Crossword Puzzle activities, including sample questions, are provided in the GBL Implementation Guide (Supplementary Materials). While in-class sessions utilized team-based formats, all digital game assets were available for voluntary, asynchronous individual use via the LMS.

### 2.6. Data Collection

#### 2.6.1. LMS Engagement Metrics

Blackboard’s built-in content access tracking system was used to record individual student access frequency for each intervention. Access counts were collected for each student (*N* = 117) across all game-based and supplementary resources and served as measures of student engagement with the learning activities. LMS engagement was operationally defined as ‘access frequency,’ representing the total number of discrete file-open events recorded by the Blackboard tracking system for each student. For example, a mean access value of 50.78 indicates the average number of times students within a specific cohort clicked to open or download the digital learning objects over the course of the semester.

#### 2.6.2. Academic Performance

Student performance was assessed using scores from Examination 2, a summative multiple-choice assessment covering the Biochemistry–Metabolism module content (33 items). Raw scores were converted to percent correct (%) and subsequently categorized into letter grade groups (D: <70%, C: 70–79%, B: 80–89%, A: 90–100%) for group-level analysis.

#### 2.6.3. Student Perception Survey

At the end of the semester, students completed a voluntary, anonymous perception survey administered through Poll Everywhere (Poll Everywhere Inc., San Francisco, CA, USA). The survey instrument included six Likert-scale items (5-point scale: 1 = Strongly Disagree to 5 = Strongly Agree) assessing perceived association of activities with learning, confidence, engagement, and enjoyment. Students were also asked to rank the available activities in order of preference, and two open-ended items solicited qualitative feedback on liked and disliked elements of the interventions. Survey participation was voluntary and anonymous; therefore, the survey analytic sample was limited to students who completed the end-of-semester survey. A total of 82 students completed the survey (response rate: 70.1%).

#### 2.6.4. Statistical Analysis

Descriptive statistics (means, standard deviations, frequencies, and percentages) were calculated for all continuous and categorical variables. Pearson correlation coefficients were computed to assess the relationship between individual game access frequency and examination performance. For group-level analysis, students were stratified by letter grade group, and mean access frequencies were calculated per group. A bubble scatter plot was generated to visually represent the relationship between mean game access frequency and grade group, with bubble size proportional to the number of students within each category. Activity ranking data were analyzed using mean rank scores. Likert-scale responses were summarized as frequencies and percentages of agree/strongly agree endorsement. Thematic content analysis was applied to open-ended qualitative responses to identify recurring themes in student feedback. All statistical analyses were conducted using Excel. A significance threshold of α = 0.05 was applied to all inferential tests.

## 3. Results

### 3.1. Participant Demographics and Academic Performance

A total of 117 first-year (P1) pharmacy students enrolled in the required Biochemistry course completed Examination 2, which served as the primary summative measure of academic performance for this study. The cohort demonstrated a mean exam score of 81.59% (*SD* = 10.75), with a median of 81.82% and scores ranging from 54.55% to 100.00%. Grade distribution was as follows: A (≥90%): *n* = 33 (28.2%); B (80–89%): *n* = 35 (29.9%); C (70–79%): *n* = 30 (25.6%); D (60–69%): *n* = 14 (12.0%); and F (<60%): *n* = 5 (4.3%). Collectively, 83.8% of students achieved a passing score of 70% or above, indicating overall satisfactory performance on the assessment (Table 1 and Table 2).

### 3.2. LMS Engagement Metrics

Student engagement with game-based and supplementary learning resources was tracked via Blackboard’s content access monitoring system for all 117 students. Mean total access across all resources had substantial individual variability in engagement patterns by student. Mean access to game-based resources specifically was 50.78 (*SD* = 35.24), while mean access to other materials was 41.39 (*SD* = 21.69) (Table 3). Among individual resources, the Pre-Class GI Diagram activity recorded the highest mean individual access count (*M* = 40.39, *SD* = 28.23), followed by the Workbook (*M* = 38.29, *SD* = 26.83). Among the game-based tools specifically, the Jeopardy: GI Vitamins and Minerals game was accessed most frequently (*M* = 5.06, *SD* = 6.87), followed by Rapid Fire: Glucose Homeostasis (*M* = 2.76, *SD* = 4.51) and the Crossword Puzzle: Amino Acids (*M* = 2.56, *SD* = 4.28). Discussion Board engagement was comparatively lower (*M* = 3.10, *SD* = 3.44).

### 3.3. Relationship Between Resource Engagement and Academic Performance

**Figure 1:**
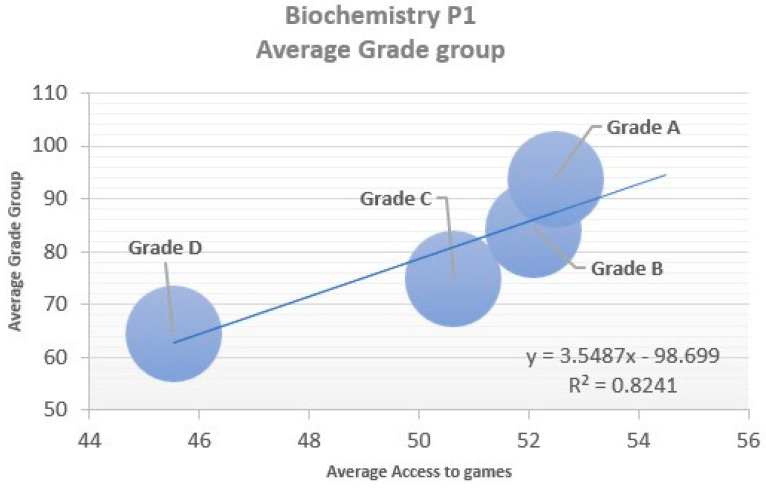
Relationship between average game access frequency and academic performance grade group among P1 pharmacy students (*N* = 117). A strong positive correlation was observed (R^2^ = 0.8241), indicating that students with higher engagement with game-based learning resources tended to achieve higher exam grades.

To examine the association between student engagement with game-based learning resources and academic performance, mean game access frequencies were calculated for each letter grade group (D, C, B, and A) and plotted against grade group categorization. A bubble scatter plot was generated to visually represent the relationship between mean game access frequency and grade group. When students were stratified by grade group, a directional pattern was observed in mean total LMS access, with students in higher-performing categories demonstrating incrementally greater overall resource engagement. (Figure 1).

However, one-way ANOVA analyses indicated that these differences did not reach statistical significance across grade groups A through D, neither for total access [*F*(3, 108) = 0.256, *p* = 0.857] nor for game-specific access [*F*(3, 108) = 0.112, *p* = 0.953]. Pearson correlation analyses similarly yielded non-significant associations between individual exam performance and total resource access (*r* = 0.086, *p* = 0.357), game access (*r* = 0.053, *p* = 0.567), Workbook access (*r* = 0.092, *p* = 0.325), and Pre-Class Diagram access (*r* = 0.086, *p* = 0.357).

At the group aggregate level, however, the scatter plot modeling mean game access frequency against grade group (D through A) yielded a positive association (R^2^ = 0.8241), indicating that approximately 82% of the variance in grade group mean was accounted for by mean game engagement across the four performance strata (Figure 1). It is important to note that this high value represents an ecological correlation based on aggregated data points and is intended for descriptive purposes only. While it illustrates a clear directional trend where higher-performing cohorts demonstrate greater average engagement, it does not imply a strong individual-level correlation, which was found to be non-significant in this study.

This group-level trend is consistent with a directional relationship between engagement and performance; however, it is important to note that this association was observed at the aggregate level and does not permit individual-level causal inference. Students achieving higher grades may reflect a broader pattern of academic engagement and self-regulated learning behaviors, of which game-based resource utilization represents one contributing component (Table 4). Nonetheless, the strength and directionality of this association are consistent with an emerging body of evidence suggesting that repeated, voluntary engagement with formative game-based tools is associated with improved academic outcomes in health professions education.

### 3.4. Student Perceptions of Game-Based Learning Activities

Of the 117 enrolled students, 82 (70.1%) completed the end-of-semester Poll Everywhere perception survey. Likert-scale results indicated broadly favorable student perceptions across all items assessed (Table 5). The majority of respondents agreed or strongly agreed that playing games helped them learn course content (*n* = 71; 95.8%, *M* = 4.20), that games and activities helped them refocus during didactic lectures (*n* = 74; 90.5%, *M* = 4.14), and that games provided a valuable opportunity to practice and test their knowledge (*n* = 77; 93.5%, *M* = 4.16). A high proportion of students also reported that the activities were enjoyable (*n* = 66; 92.4%, *M* = 4.21), and 82.9% indicated that games helped them develop confidence in the course material (*n* = 76; *M* = 3.80).

Perceptions of collaborative use were more heterogeneous: only 46.8% of respondents agreed or strongly agreed that they used games for group work (*M* = 2.95), suggesting that while some students utilized activities collaboratively, the majority engaged with them as individual self-study tools. With respect to the Discussion Board, 67.6% of respondents considered it a useful resource (*n* = 68; *M* = 3.41); however, actual participation was more limited, with only 48.7% indicating they actively contributed to or engaged with the board (*n* = 78; *M* = 2.88), suggesting a gap between perceived utility and actual engagement.

### 3.5. Activity Preference Results

Of the 82 survey respondents, 65 (79.3%) completed the activity preference ranking item. Students ranked nine available activities in order of preference, with rank 1 indicating the most preferred (Table 6). The Jeopardy game was the most preferred activity overall (mean rank = 2.49), receiving the highest number of first-place votes (*n* = 23; 35.4% of respondents). Poll Everywhere ranked second (mean rank = 3.31; 12 first-place votes), followed by the Workbook (mean rank = 3.85; 13 first-place votes). Glucose Homeostasis Puzzle ranked fourth (mean rank = 5.12), followed by the Rapid Fire game (mean rank = 5.20), and Crossword Puzzle (mean rank = 5.75).

### 3.6. Qualitative Findings: Student Feedback

Open-ended survey responses were analyzed thematically. A total of 57 students provided feedback on activities they liked, and 61 provided feedback on aspects they disliked.

A. Positive themes clustered into five primary categories:

(1) *Engagement and interactivity*—students frequently described activities as fun, energizing, and competitive, with many stating that games provided a welcome break from traditional didactic instruction (*“They re-energized me”; “Having a break from just didactic lectures, they were fun”*);

(2) *Knowledge reinforcement*—respondents indicated that games facilitated active recall and exam preparation (*“It’s easier to play games like this than traditional studying”*);

(3) *Workbook utility*—the Workbook was widely praised as a practical concurrent study guide (*“The workbook was super helpful!!”*);

(4) *Collaborative team-based learning*—Jeopardy was specifically cited for promoting peer collaboration (*“I enjoyed doing the games as a class in teams”*); and

(5) *Instructor enthusiasm and preparation*—students frequently acknowledged faculty energy as a motivating factor.

B. Constructive topics were also identifiable. Concerns included: activities being clustered at the end of class sessions rather than distributed throughout; the absence of a Workbook answer key; the pace of the Rapid Fire game being perceived as too fast; and requests for more video animations and multimedia content.

## 4. Discussion

This study investigated the effect of an integrated suite of GBL and active engagement strategies on academic performance and student perceptions in a P1 pharmacy biochemistry course. Overall, findings suggest that low-cost, faculty-developed GBL tools are feasible, well-received, and associated with positive directional trends in performance within this foundational context.

### 4.1. Academic Performance and Engagement

At the group aggregate level, a positive association was observed between mean game-based resource access and grade group performance (R^2^ = 0.8241), with students in higher-performing grade categories consistently demonstrating greater mean engagement with available game resources. This pattern aligns with evidence documenting positive associations between game-based engagement and learning outcomes in health professions education [15,16].

However, at the individual level, correlation analyses between game access frequency and exam performance were non-significant, and this pattern held across all other resources examined. These findings underscore an important methodological consideration: the group-level aggregate trend reflects a meaningful pattern when comparing mean student profiles across performance but does not translate into a simple linear individual-level relationship. This distinction is critical for responsible interpretation of the data. Students who achieve higher grades are likely to represent a cluster of self-regulatory behaviors, of which GBL engagement is one observable indicator, rather than a singular causal driver of performance. These findings suggest that GBL engagement should be viewed through the lens of social-cognitive theories of self-regulated learning, being a mediational process where students actively monitor and control their learning to meet specific goals [17]. Similarly, research has demonstrated that successful learners move through cyclical phases of forethought, performance, and self-reflection [18]. In this context, the GBL activities provided a high-utility platform for these students to engage in the performance and monitoring phases of their study cycle, effectively transforming their effort into academic achievement [17,18]. Academic self-regulation, prior knowledge, cognitive load management, and intrinsic motivation all moderate the relationship between resource engagement and achievement in ways that a single access frequency metric cannot fully capture. Importantly, the non-significant individual-level correlations should not be interpreted as evidence that GBL is ineffective; rather, they suggest that the benefits of GBL may be distributed across diverse learner profiles and are more richly captured through perceptual, affective, and self-regulatory indicators, precisely the dimensions assessed by the survey instrument.

The discrepancy between the strong aggregate-level trend and the weak individual-level correlations highlights a common phenomenon in educational research known as aggregation bias [19]. By averaging data into performance groups, individual-level ‘noise’ is reduced, surfacing a clear behavioral profile of the ‘typical’ student in each grade category. However, to avoid the ecological mistake, these findings must not be used to predict an individual student’s grade based solely on their access frequency. Instead, this trend reinforces the interpretation that high-achieving students, as a group, leverage GBL tools as part of a broader suite of self-regulatory behaviors.

A limitation of utilizing LMS access data is that access frequency serves as a behavioral representation of engagement intent rather than a direct measure of learning depth or task completion. Blackboard metrics record the initiation of an interaction but do not capture time-on-task, the quality of cognitive processing, or whether a student completed a game in its entirety. However, the strong positive association between access frequency and academic performance suggests that these counts are not merely ‘noise’ but represent a meaningful signal of student effort. Repeatedly returning to GBL resources is a hallmark of self-regulated learners who utilize available tools for iterative self-testing and knowledge reinforcement [17,18]. While future research using more granular SCORM data (e.g., in-game scores or time-per-slide) could provide deeper insights, the current access metrics provide a validated, objective indicator of the differential resource utilization patterns between high- and low-achieving students.

### 4.2. Student Perceptions and Activity Preferences

Student perceptions of GBL were overwhelmingly positive across domains. Survey data revealed strikingly positive perceptions of the GBL and active learning activities across all core domains assessed. The highest endorsement was observed for perceived utility for practice and knowledge testing, followed by enjoyment, benefit for refocusing during didactic lectures, and perceived learning benefit. A strong majority (82.9%) reported that games helped build confidence in the material. These findings align with constructivist and active learning frameworks, which suggest that learner engagement, emotional investment, and perceived relevance are critical mediators of knowledge construction [20,21]. The high ratings across engagement and learning utility dimensions suggest that the multimodal design of the curriculum intervention, combining competitive team games, individual asynchronous tools, and structured workbook exercises, was effective in addressing diverse learning preferences within the cohort. Particularly notable is the consistency of high endorsement across items mapping onto distinct pedagogical functions: refocusing attention during lectures (in-class active learning), knowledge testing (formative self-assessment), and confidence building (affective dimension of learning). This pattern suggests that the GBL suite was perceived as multi-dimensionally valuable rather than a single-purpose novelty, a finding that strengthens the case for its continued integration. These results corroborate findings from analogous studies in health professions education, where students consistently value active, low-stakes learning formats aligned with high-stakes course content [22,23].

Certain activities have a preference for pedagogical value. Activity preference rankings among 65 respondents identified Jeopardy as the most preferred activity, followed by Poll Everywhere and the Workbook. These preferences are pedagogically informative. Jeopardy’s position as the top-ranked activity is consistent with its design features: it combines competitive team dynamics, immediate feedback, structured recall practice, and high energy, elements associated with deep encoding and intrinsic motivation in the GBL literature [24]. However, it is important to note that game-based learning tools must remain flexible to accommodate diverse learning preferences. Our survey data revealed that only 46.8% of respondents used the games for group work, indicating that over half the cohort engaged with these tools as individual self-study resources. This suggests that the pedagogical value of the intervention lies not only in its collaborative potential but also in its utility as a structured, individual formative assessment tool for students who prefer independent learning or lack access to study groups.

The qualitative data reinforces this interpretation, with 36.8% of positive open-ended responses referencing games or Jeopardy specifically in terms of fun, engagement, and competitive stimulation (“*Jeopardy was so helpful*”; “*Having a break from just didactic lectures, they were fun*”). Additionally, the strong ranking of the Workbook (an instructor-designed, fill-in-the-blank concurrent learning tool) is a noteworthy finding. Despite not being per se a “game,” it received more first-place votes than Poll Everywhere and ranked above Rapid Fire and the Crossword Puzzle. This suggests that students value structured, self-paced practice that runs concurrently with lecture content, and that perceived utility may be as important as entertainment value in determining preference. This is consistent with Bloom’s taxonomy-informed instructional design principles, where activities that directly scaffold knowledge application in alignment with content delivery are perceived as highly relevant [25].

The Discussion Board (using Padlet) presented a noteworthy paradox. While 67.6% of students considered it a useful resource, only 48.7% reported actively participating by posting, responding, or engaging with content. This utility-participation gap is a well-documented phenomenon in asynchronous digital learning environments [26], where students may perceive the value of peer-generated content without feeling sufficient intrinsic motivation or social accountability to contribute actively. The lower participation rate may also reflect platform unfamiliarity, uncertainty about expectations, or a preference for passive consumption over active contribution within an already demanding first-year curriculum. Future implementations could address this gap through structured discussion prompts, faculty-seeded questions, or explicit participation expectations to lower the barrier to initial engagement.

The most prominent concern to emerge from qualitative analysis was the pace of the Rapid Fire: Glucose Homeostasis game. Some students found the time pressure stressful rather than motivating. While time-pressured recall activities are well-supported in the cognitive psychology literature as facilitators of memory consolidation through desirable difficulty [27], they may not be appropriate as the sole format for all content types or all learners. Offering a practice mode without time pressure alongside the competitive version could extend the tool’s accessibility without diminishing its success for students who benefit from the challenge.

Students additionally expressed a preference for activities distributed throughout the class session rather than concentrated at the end of the lecture and requested more video and animation content. These preferences reflect students’ awareness of their own attentional regulation and suggest that cognitive fatigue in the latter portions of long didactic sessions may limit the pedagogical effect of activities placed there, a finding with direct implications for session planning.

A central contribution of this study is its focus on a first-year foundational basic science course, a context that has been systematically underrepresented in the pharmacy education GBL literature. The biochemistry course examined here serves a gatekeeping function within the PharmD curriculum, where mastery of its content is a prerequisite to meaningful engagement with pharmacokinetics, pharmacodynamics, medicinal chemistry, and clinical therapeutics in subsequent years. Students who struggle to engage with foundational biochemical concepts in the P1 year do not simply underperform on a single examination; they enter subsequent courses with conceptual gaps that compound over time, increasing the risk of academic difficulty, remediation, and attrition from the program. The high-stakes nature of this foundational stage makes it a priority target for evidence-based pedagogical intervention, yet it has historically received less attention than the clinical courses that follow it. The present findings suggest that GBL and active engagement strategies are not only feasible in this context but are perceived by students as genuinely valuable for learning, practice, and confidence-building, precisely the outcomes most needed at the foundational stage. Faculty and curriculum designers in pharmacy programs should therefore consider the P1 basic science curriculum as a high-priority site for active learning investment, recognizing that improvements in engagement and mastery at this level carry multiplicative benefits across the entire professional curriculum.

### 4.3. Implications for Practice

As for the implications for Pharmacy education, collectively, these findings support the integration of GBL and multimodal active learning within pharmacy biochemistry education as a strategy to enhance student engagement, affective learning experiences, and perceived learning utility.

In summary, several specific practice implications emerge from these data:

1. Prioritize in-class team-based competitive formats. Jeopardy-style games with collaborative team structures generate the highest perceived value and engagement, consistent with social interdependence theory [28]. These should be retained and expanded in future course iterations.
2. Retain and scaffold workbook-style concurrent learning tools. The strong showing of the Workbook in preference rankings affirms that structured, instructor-designed practice materials aligned with lecture content remain highly valued by students even within a GBL framework.
3. Re-evaluate high-anxiety activity designs. Redesign fast games or replace them with voluntary or anonymous performances.
4. Distribute activities across the session. GBL and active learning activities should be intentionally embedded at multiple points throughout the lecture rather than concentrated at the end, reducing cognitive fatigue effects and sustaining engagement across the full session.
5. Expand multimedia integration. Incorporating targeted video clips and animations aligned with complex pathway content would address student requests and accommodate visual learning preferences, further enriching the multimodal learning environment.
6. Support Discussion Board engagement through structured facilitation. To bridge the utility-participation gap observed with Padlet, future implementations should incorporate structured prompts, faculty seeding, and clear participation expectations.

It is worth noting that the value of the presented model lies in its pedagogical portability rather than its technical platform. While the specific tools used in this study have faced ‘digital decay’ or platform transitions, the underlying mechanics (immediate feedback, competitive engagement, and collaborative problem-solving) are platform-agnostic [29,30]. The strong correlation observed between game access and academic performance (Figure 1) suggests that student success was driven by the process of engagement with the content, not the specific digital vehicle. By providing the structural blueprints and sample content in the appendices, this study offers a replicable framework that can be adapted to various technological ecosystems, ensuring that the benefits of GBL remain accessible despite the rapid evolution of educational software.

### 4.4. Limitations and Future Directions

Several limitations of this study warrant acknowledgment. First, the study employed a single-group, cross-sectional design without a control comparator, precluding causal attribution of performance outcomes to the GBL intervention. Our primary aim was to document the implementation and explore the associations between engagement and performance in an authentic, real-world educational setting [31]. Because this study employed a single-group, cross-sectional design without a randomized or closely matched comparison group, causal inference is limited. These results should be interpreted as evidence of association and engagement patterns within this specific cohort, rather than as definitive proof that the intervention caused the observed performance differences relative to traditional instruction. While a strong positive association was observed between mean game access frequency and grade group, we cannot rule out the influence of confounding variables such as student self-regulation, prior academic achievement, or concurrent study habits. While a randomized controlled trial is often considered the gold standard for evaluating educational interventions, this study utilized a single-group design to ensure equitable access to all learning resources. In professional pharmacy education, withholding a potentially beneficial intervention from a subset of students is often viewed as a barrier to student success and a violation of the institutional duty to provide optimal training for all learners. Furthermore, historical cohorts at this institution do not serve as pure control groups, as they were not entirely devoid of active learning elements. However, those previous interventions were sporadic, non-systematic, and lacked the multimodal integration utilized in the current study. Consequently, this investigation serves as a feasibility and association evaluation, establishing the practical integration of low-cost, faculty-developed game-based learning tools within a foundational pharmacy biochemistry curriculum and measuring their incremental effect over the traditional baseline standard of instruction. Second, LMS access counts represent a proxy measure of engagement that does not capture depth of engagement, time-on-task, or quality of interaction with the material. Third, the survey was voluntary and anonymous, introducing potential non-response bias; notably, the “Want activities in future” item yielded only seven valid responses, limiting the interpretability of that item. Fourth, the study was conducted in a single course at a single institution, limiting generalizability to other programs or populations. Fifth, student characteristics, including prior academic achievement, learning styles, and self-regulatory capacity, were not systematically assessed and may represent important confounders in the engagement-performance relationship. Additionally, the study focused on a single foundational basic science course within the P1 year; future research should examine whether similar GBL interventions yield comparable engagement and performance outcomes across other foundational courses, including physiology, immunology, medicinal chemistry, and pharmacokinetics, that collectively constitute the basic science foundation of the PharmD curriculum.

A significant limitation with respect to study replicability concerns the availability of the game-based learning resources described herein. The majority of games developed for this study were created using WiscOnline, a free educator-developed platform that has since undergone a structural transition to WisTechOpen. During this migration, user-generated games central to this intervention were subject to institutional review and were not preserved in an accessible form. Consequently, the specific game-based tools described in this manuscript cannot be directly reproduced or deployed in future cohorts using the same platform and files. While the pedagogical design principles and WiscOnline/WisTechOpen framework remain applicable, future implementations would require the reconstruction of game content from the ground up. This limitation highlights a broader vulnerability in GBL research: the dependence on third-party platforms whose continuity, content policies, and data retention practices are outside the control of individual faculty or institutions.

A second replicability limitation concerns the institutional transition from Blackboard to Canvas at the University of Houston in 2024. The engagement metrics reported in this study, including individual resource access counts, game interaction frequencies, and content tracking data, were collected through Blackboard’s native analytics infrastructure during the Fall 2023 semester. Canvas employs a fundamentally different data architecture and engagement tracking methodology, meaning that equivalent metrics cannot be collected in an identical manner in subsequent cohorts. Direct longitudinal comparisons of LMS engagement data across pre- and post-migration cohorts would therefore require careful methodological reconciliation and should be interpreted with caution. Future studies conducted within a Canvas environment may benefit from the platform’s analytics capabilities, including time-on-task data and page-view tracking, which could provide richer engagement metrics than those available through Blackboard’s access count system.

A final notable limitation of this study concerns the long-term accessibility of the game-based learning resources described herein. During an institutional platform migration from WiscOnline to WisTechOpen, user-generated game content central to this intervention was subject to institutional review and was not preserved in an accessible form. Consequently, the specific game-based tools described in this manuscript cannot be directly reproduced or deployed in future cohorts using the same platform and files. This experience highlights a broader vulnerability in GBL research: the dependence on third-party platforms whose continuity, content policies, and data retention practices lie outside the control of individual faculty or institutions. Future implementations would require reconstruction of game content from the ground up, and researchers are strongly encouraged to export and archive SCORM packages locally at the time of creation to protect against institutional or platform-level transitions.

## 5. Conclusions

The findings of this study underscore the value of integrating GBL and multimodal active learning into pharmacy biochemistry education. The practical accessibility of digital tools combined with seamless LMS integration via SCORM positions this approach as scalable and feasible for broad adoption without requiring specialized technical infrastructure, a meaningful consideration for faculty seeking to enhance student engagement within existing resource constraints.

Future studies should employ experimental or quasi-experimental designs with matched control groups to enable stronger causal inference regarding GBL’s effect on performance. Research examining the relationship between GBL engagement quality, not merely frequency, and self-regulatory learning behaviors would meaningfully advance the field. Additionally, investigation of GBL scalability across different health professions educational contexts, including nursing, medicine, and allied health programs, and across diverse or non-traditional student populations, would extend the generalizability of these findings and inform broader adoption strategies.

## Figures and Tables

**Table 1:** Participant Demographics.

Characteristic	*n*	%
**Total enrolled**	117	100%
**Gender**		
** Female**	61	52.1%
** Male**	56	47.9%
**Age (years)**		
** Range**	21–29	—
** Mean (*SD*)**	24.0	—
**Year in program**		
** First year (P1)**	117	100%
**Survey respondents**	82	70.1%

**Table 2:** Academic Performance Summary.

Performance Metric	Value
***N***	117
**Mean score (%)**	81.59
**Standard deviation**	10.75
**Median score (%)**	81.82
**Minimum score (%)**	54.55
**Maximum score (%)**	100.00
**Grade Distribution**	
** A (≥90%)**	33 (28.2%)
** B (80–89%)**	35 (29.9%)
** C (70–79%)**	30 (25.6%)
** D (60–69%)**	14 (12.0%)
** F (<60%)**	5 (4.3%)
**Passing (≥70%)**	98 (83.8%)

**Table 3:** LMS Engagement Metrics by Resource.

Resource	Mean Accesses	*SD*	Min	Max
**Games**				
Pre-Class Diagrams	40.39	28.23	2	205
Jeopardy: GI Vitamins and Minerals	5.06	6.87	0	37
Rapid Fire: Glucose Homeostasis	2.76	4.51	0	28
Crossword Puzzle: Amino Acids	2.56	4.28	0	26
**Workbook**	38.29	26.83	2	1164
**Discussion Board (Padlet)**	3.10	3.44	0	18

**Table 4:** Mean Game Access by Grade Group.

Grade Group	*n*	Mean Game Access	*SD*	Mean Total Access	*SD*
A (≥90%)	33	53.94	45.52	199.76	156.35
B (80–89%)	35	51.17	34.20	197.89	123.27
C (70–79%)	30	50.17	29.45	182.37	94.64
D (60–69%)	14	47.79	29.42	170.50	114.93
F (<60%)	5	39.20	10.66	125.60	74.81
*F* statistic		*F*(3, 108) = 0.112	*p* = 0.953	*F*(3, 108) = 0.256	*p* = 0.857

**Table 5:** Student Perception Survey Results.

Survey Item	*n*	*M*	*SD*	Agree/Strongly Agree
Activities were fun/enjoyable	66	4.21	0.85	92.4%
Games helped learn the course content	71	4.20	0.65	95.8%
Games provided practice opportunities	77	4.16	0.78	93.5%
Games helped refocus during lectures	74	4.14	0.88	90.5%
Games built confidence in material	76	3.80	0.98	82.9%
Discussion board was a useful resource	68	3.41	1.14	67.6%
Used games for group work	77	2.95	1.41	46.8%
Participated in the discussion board	78	2.88	1.38	48.7%

Likert scale: 1 = Strongly Disagree, 5 = Strongly Agree. Items ordered by Agree/Strongly Agree endorsement rate.

**Table 6:** Activity Preference Rankings.

Rank	Activity	*n*	Mean Rank	*SD*	1st-Place Votes	% 1st Place
1	Jeopardy Game	65	2.49	1.82	23	35.4%
2	Poll Everywhere	65	3.31	1.95	12	18.5%
3	Workbook	65	3.85	2.20	13	20.0%
4	Glucose Homeostasis Puzzle	65	5.12	1.88	1	1.5%
5	Rapid Fire Game	65	5.20	2.25	2	3.1%
6	Crossword Puzzle	65	5.75	1.75	0	0.0%

Lower mean rank = higher preference. Rank 1 = most preferred.

## Data Availability

The data presented in this study are available on request from the corresponding author due to privacy restrictions related to student records and limitations of third-party platforms. Certain methodologies and digital tools described are no longer accessible in their original format due to platform migrations and service changes (e.g., WiscOnline and Jamboard), which may hinder direct replication.

## Supplementary Materials

The following supporting information can be downloaded at: https://www.mdpi.com/article/10.3390/pharmacy14040104/s1, File S1: Game-Based Learning (GBL) Summary Implementation Guide for Pharmacy.
