# Supplementary material for: Boosting Pharmacy Foundational Science Education Through Game-Based Learning and Active Engagement Strategies
Source: Pharmacy (Basel). 2026 Jul 9;14(4):104. doi: 10.3390/pharmacy14040104 (PMC13415470; doi:10.3390/pharmacy14040104)

S1: Game-Based Learning Summary Implementation Guide for Pharmacy (Examples)

This guide outlines the pedagogical framework and structural design of the GBL interventions utilized in this study. While the original digital objects were authored in WiscOnline (now WisTechOpen), the following blueprints are platform-agnostic and can be replicated using any contemporary GBL tool (e.g., Kahoot!, Quizizz, Hot Potatoes, or H5P).

1. Jeopardy: GI Vitamins & Minerals

Core Learning Objective: To reinforce factual recall of vitamin/mineral functions and the conceptual understanding of their absorption and clinical deficiency syndromes.

Game Structure and Rules:

- **Format:** Team-based competitive play for in-classroom competition with 4 groups. Study group competitions → 3 groups (3-4 students). The third option is playing "solo".
- **Grid:** A 4x5 matrix with five categories and increasing point values (100–500).
- **Rules:** Teams rotate turns selecting categories. A "buzz-in" system is used for steals. Correct answers gain points; incorrect answers result in no point change to encourage participation.

- **Duration:** In class → 20-30 minutes, including a 10-minute “within-game” debrief.

Sample Biochemistry Content Questions:

1. **100 pts:** This vitamin requires Intrinsic Factor for absorption in the terminal ileum. *(Ans: Vitamin B12)*
2. **200 pts:** Scurvy is the classic clinical manifestation of a deficiency in this water-soluble antioxidant. *(Ans: Vitamin C)*
3. **300 pts:** These four vitamins are classified as fat-soluble and require dietary lipids for optimal absorption. *(Ans: Vitamins A, D, E, and K)*
4. **400 pts:** This mineral serves as the central coordinating ion in the heme group of hemoglobin. *(Ans: Iron)*
5. **500 pts:** Wernicke-Korsakoff syndrome is most commonly associated with a deficiency in this B-vitamin, often seen in chronic alcohol use. *(Ans: Thiamine/B1)*

Type of Feedback:

- **Immediate:** The digital platform provides an instant "Correct/Incorrect" visual and auditory cue.
- **Instructional:** The facilitator provides a 1–2 minute "micro-lecture" after each high-value question to clarify biochemical mechanisms or clinical relevance.

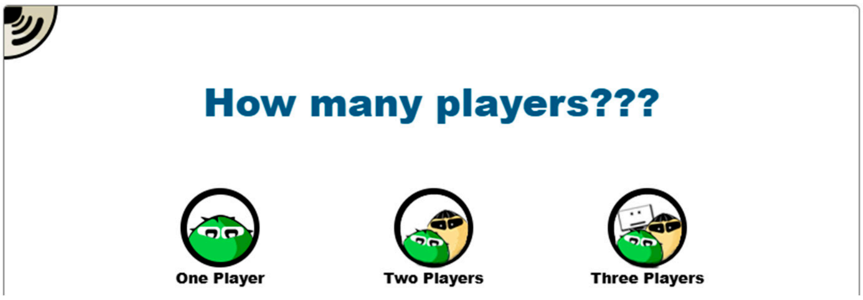

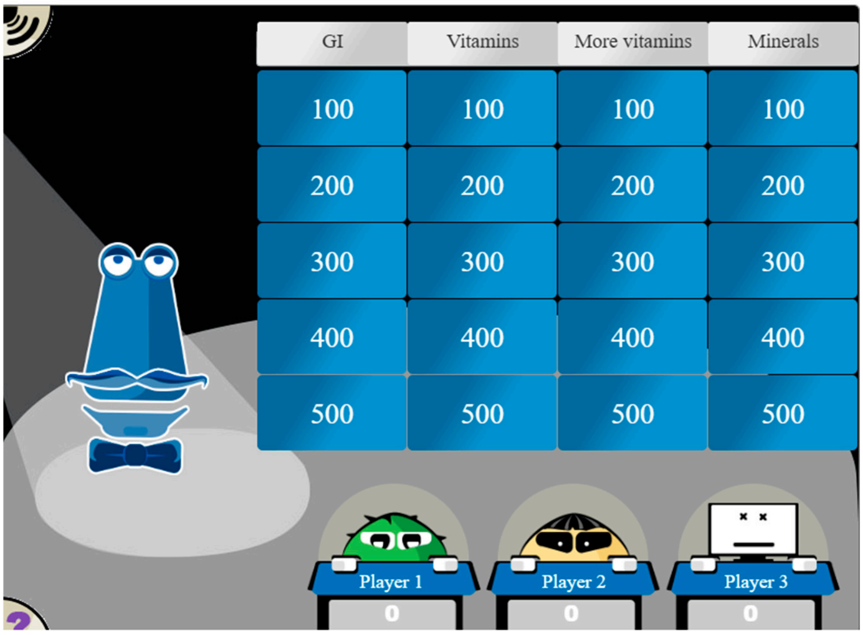

2. Rapid Fire: Glucose Homeostasis

Core Learning Objective: To improve the speed and fluency of recall for key hormones, enzymes, and metabolic pathways involved in blood glucose regulation.

Game Structure and Rules:

- **Format:** Individual or small-group "drill" style, when studying in groups.

- **Sequence:** 20 + short-answer questions delivered in slow or rapid succession (student choose speed).
- **Rules:** Students have ~15 seconds per item. The goal is to achieve the highest "streak" of correct answers.
- **Duration:** 10–15 minutes as a high-energy "warm-up" or "wrap-up" activity.

Sample Biochemistry Content Questions:

1. Which pancreatic cells are responsible for the secretion of insulin? *(Ans: Beta cells)*
2. Does glucagon stimulate or inhibit the process of glycogenolysis? *(Ans: Stimulate)*
3. What is the primary storage polysaccharide of glucose in the human liver? *(Ans: Glycogen)*
4. Name the metabolic pathway that creates glucose from non-carbohydrate precursors like glycerol or lactate. *(Ans: Gluconeogenesis)*
5. Which hormone is the primary antagonist to insulin in the regulation of blood sugar? *(Ans: Glucagon)*

Type of Feedback:

- **Automated:** The platform displays the correct answer immediately after the timer expires, allowing for instant self-correction.

- **Performance Tracking:** Students receive a final score or "accuracy percentage" at the end of the session to track their own progress over time.

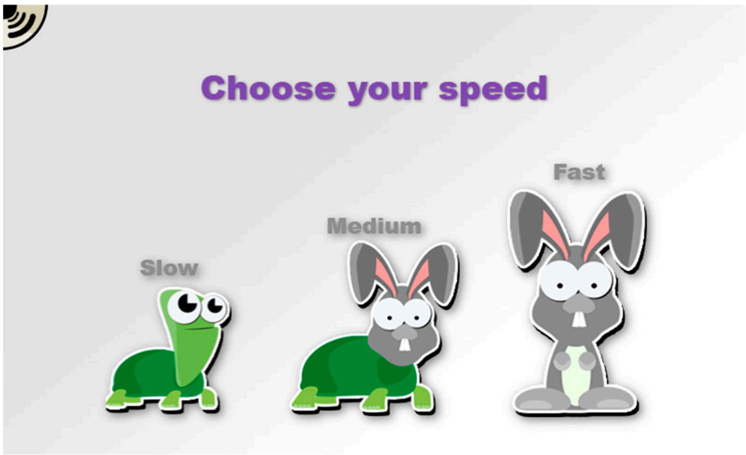

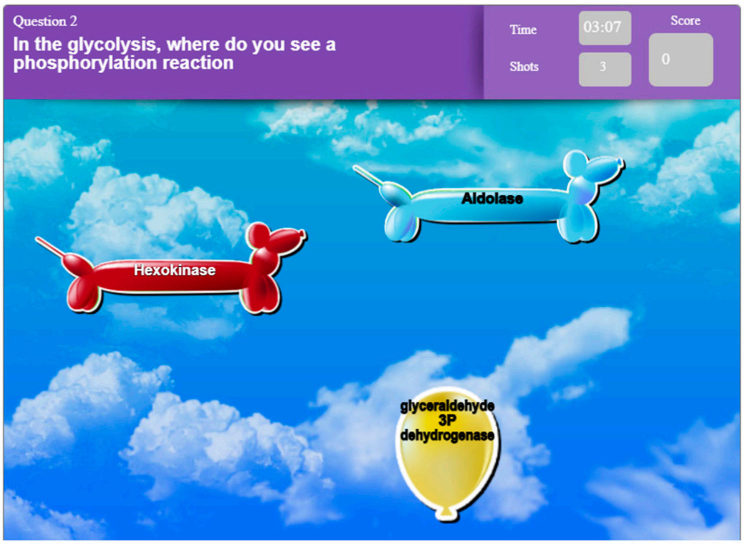

3. Crossword Puzzle: Amino Acids

Core Learning Objective: To reinforce the terminology, structural classifications, and chemical properties of the 20 standard proteinogenic amino acids.

Game Structure and Rules:

- **Format:** Collaborative pairs or individual puzzle-solving.

- **Grid:** A standard crossword layout where clues describe the properties of specific amino acids.
- **Rules:** Students use their notes or memory to complete the grid. The activity is low-stakes and untimed to encourage deep reflection.
- **Duration:** 20 minutes of in-class group activity or asynchronous work.

Sample Biochemistry Content Clues:

1. **Across:** The only amino acid that lacks a chiral center. *(Ans: Glycine)*
2. **Down:** A sulfur-containing amino acid capable of forming disulfide bridges. *(Ans: Cysteine)*
3. **Across:** A basic amino acid that carries a positive charge at physiological pH. *(Ans: Lysine or Arginine)*
4. **Down:** The category of amino acids that cannot be synthesized de novo and must be obtained via diet. *(Ans: Essential)*
5. **Across:** The specific covalent bond that links the carboxyl group of one amino acid to the amino group of another. *(Ans: Peptide)*

Type of Feedback:

- **Visual/Structural:** The crossword grid provides inherent feedback; if a word does not fit the letter count or intersections, the student knows an error has occurred.
- **Summative:** A completed answer key is provided via the LMS after the session for final verification.

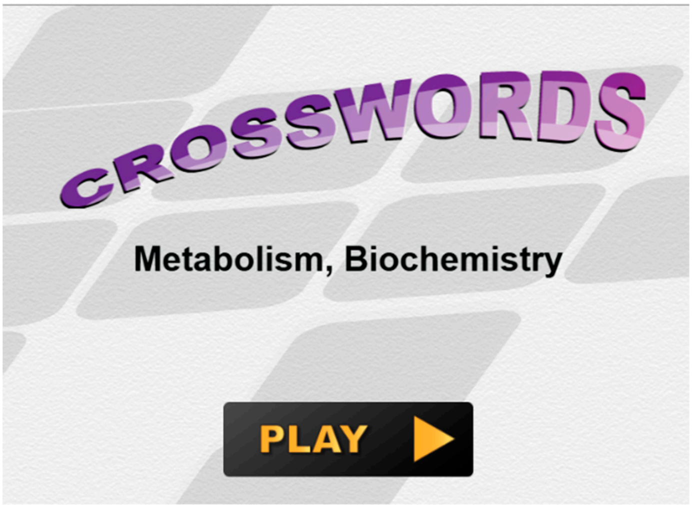

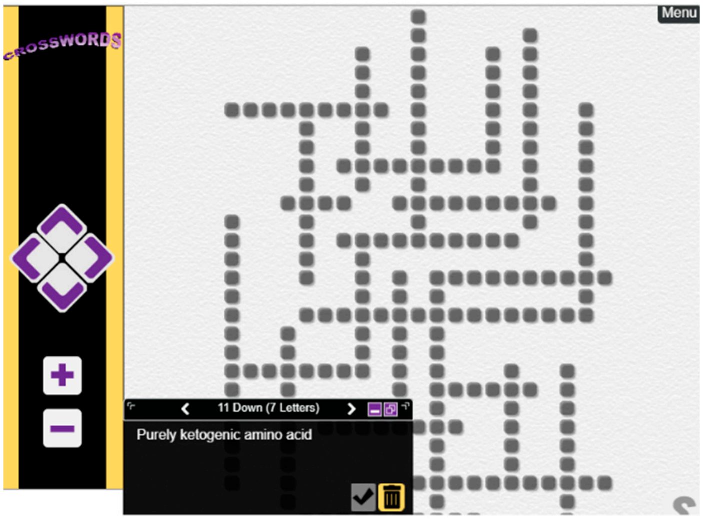

Supplement: Supplementary file 1 [file pharmacy-14-00104-s001.zip › pharmacy-4382892-supplementary.pdf]
